# Hybrid-Recursive-Refinement Network for Camouflaged Object Detection

**DOI:** 10.3390/jimaging11090299

**Published:** 2025-09-02

**Authors:** Hailong Chen, Xinyi Wang, Haipeng Jin

**Affiliations:** School of Computer Science and Technology, Harbin University of Science and Technology, Harbin 150080, China; 2320400021@stu.hrbust.edu.cn (X.W.); hpjin@hrbust.edu.cn (H.J.)

**Keywords:** camouflaged object detection, feature fusion strategy, complementary information strategy, edge refinement

## Abstract

Camouflaged object detection (COD) seeks to precisely detect and delineate objects that are concealed within complex and ambiguous backgrounds. However, due to subtle texture variations and semantic ambiguity, it remains a highly challenging task. Existing methods that rely solely on either convolutional neural network (CNN) or Transformer architectures often suffer from incomplete feature representations and the loss of boundary details. To address the aforementioned challenges, we propose an innovative hybrid architecture that synergistically leverages the strengths of CNNs and Transformers. In particular, we devise a Hybrid Feature Fusion Module (HFFM) that harmonizes hierarchical features extracted from CNN and Transformer pathways, ultimately boosting the representational quality of the combined features. Furthermore, we design a Combined Recursive Decoder (CRD) that adaptively aggregates hierarchical features through recursive pooling/upsampling operators and stage-wise mask-guided refinement, enabling precise structural detail capture across multiple scales. In addition, we propose a Foreground–Background Selection (FBS) module, which alternates attention between foreground objects and background boundary regions, progressively refining object contours while suppressing background interference. Evaluations on four widely used public COD datasets, CHAMELEON, CAMO, COD10K, and NC4K, demonstrate that our method achieves state-of-the-art performance.

## 1. Introduction

Camouflage, whether occurring naturally or through artificial means, serves as a strategy to reduce object visibility by imitating environmental attributes such as color, texture, and morphology [1]. COD addresses the intricate problem of detecting and localizing objects that blend seamlessly into cluttered or visually similar backgrounds. This task holds considerable importance in various real-world scenarios, including medical image analysis for lesion segmentation [2,3], surface defect inspection in industrial settings [4], and automated pest detection systems in precision agriculture [5].

Early COD methods based on handcrafted features [6,7] exhibited poor generalization and were highly susceptible to background interference. The advancement of CNN architectures has been instrumental in driving progress in camouflaged object detection, primarily due to their proficiency in extracting discriminative visual features [8,9,10]. Nevertheless, the locality constraint of CNN receptive fields limits their capacity to model global context, often resulting in incomplete segmentation or inaccurate localization. To address these shortcomings, Transformer-based models have recently gained traction in visual tasks, leveraging self-attention mechanisms to effectively capture long-range dependencies. Their powerful global representation capabilities have opened new avenues for tackling the challenges inherent in COD [11,12,13]. Nevertheless, directly aggregating features with large semantic discrepancies often leads to the loss of subtle but crucial local information, making it difficult to precisely delineate camouflaged object boundaries. While the aforementioned approaches have achieved varying degrees of success, they still face inherent limitations in complex and diverse camouflaged scenarios.

**Figure 1:**
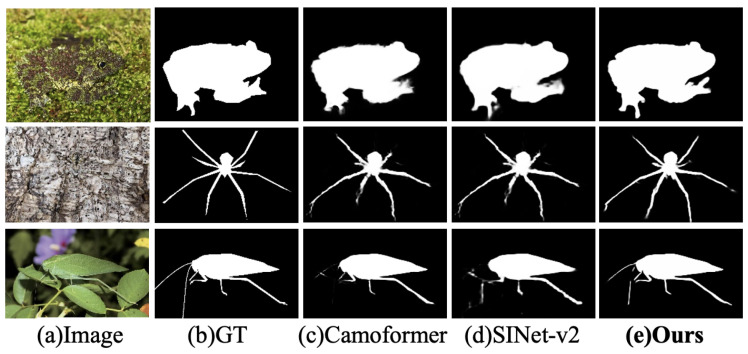
Comparison with other methods. It can be observed that both the CNN-based SINet-v2 and the Transformer-based CamoFormer exhibit challenges in achieving complete target detection and accurately capturing edge details.

Some recent studies have explored hybrid architectures that fuse CNN and Transformer encoders, showing promising results in vision tasks [14,15]. However, effectively integrating their complementary advantages remains a key challenge. Additionally, the features extracted by the encoders are inherently hierarchical: shallow high-frequency features are rich in spatial details but prone to noise, whereas deep low-frequency features contain more semantic information but often lose fine spatial cues. Therefore, designing a decoder that can effectively process and integrate multi-level features—fusing semantic and spatial information while suppressing noise—is essential [16]. Moreover, the substantial visual similarity between camouflaged objects and their surrounding backgrounds poses a critical challenge in COD, often leading to foreground regions being mistakenly identified as background. Ignoring background modeling can lead to incomplete object detection or inaccurate boundary localization. To address this, researchers have explored solutions such as texture-aware modeling [17,18] and edge-guided features [19,20]. As illustrated in Figure 1, both CNN-based methods like SINet-v2 [21] and Transformer-based methods like CamoFormer [22] still suffer from limitations such as incomplete object detection and poor edge localization.

Motivated by the aforementioned insights, we introduce a Hybrid Recursive Refinement Network (HRRNet), which comprises three cohesively designed components. Initially, multi-scale features extracted from the CNN and Transformer encoders are processed by an HFFM, which aligns and integrates the dual-branch representations through channel-wise interaction and fusion, effectively bridging the semantic gap and promoting robust feature integration. Second, to better decode the hierarchical features extracted by the encoders, we propose a CRD, which comprises three submodules: Recursive Pooling, Recursive Upsampling, and Recursive Aggregation. These modules iteratively refine and fuse multi-scale information to generate high-quality decoding features. Finally, an FBS module is proposed to refine object boundaries and effectively reduce background distractions. This module alternately attends to foreground and background regions, emphasizing ambiguous regions that are prone to being confused. By adaptively refining and re-integrating misclassified features, FBS produces more complete and accurately bounded predictions. Our contributions are as follows:

- We introduce a new hybrid architecture, termed HRRNet, which integrates features extracted by CNN and Transformer encoders through an HFFM. This module performs decoupled channel-wise fusion to exploit the complementary strengths of both feature extractors effectively.
- We design a CRD that adaptively adjusts the feature fusion strategy based on the feature hierarchy. By fusing low-level detail with high-level semantics, the decoder effectively estimates the probable locations of camouflaged targets.
- We present an FBS module, which selectively focuses on either foreground or background regions to iteratively refine segmentation results, enabling accurate identification of camouflaged object boundaries.

The remainder of this paper is organized as follows. Section 2 reviews related work on camouflaged object detection and complementary information strategy. Section 3 presents the proposed framework and its key components. Section 4 first describes the experimental setup, datasets, and evaluation metrics. It then analyzes the experimental results, including comparisons with state-of-the-art methods as well as ablation studies. Finally, Section 5 concludes the study and outlines possible directions for future research.

## 2. Related Work

### 2.1. Camouflaged Object Detection

The emergence of large-scale benchmark datasets, including CAMO [23], COD10K [24], and NC4K [25], has markedly accelerated progress in deep learning approaches for COD. CNNs have been widely adopted as the foundational architecture for visual recognition and segmentation tasks, owing to their capacity for processing inputs of varying dimensions and extracting multi-level feature representations. As a result, most current COD approaches are built upon CNN-based models. For instance, some biologically inspired methods simulate predator perception mechanisms (e.g., SINet-v2 [21]) or leverage scene-level transformations (e.g., ZoomNet [26]) to capture camouflaged targets. Recent studies have proposed multi-task learning frameworks that improve prediction accuracy by introducing auxiliary supervision signals, including edge detection [27], scribble-based annotations [28], and edge reconstruction strategies such as FEDER [8]. Despite the notable performance of CNN-based models, their inherently constrained receptive fields limit the ability to capture long-range dependencies across the entire image, often resulting in suboptimal delineation of camouflaged objects with incomplete structural recovery. Given the strength of Transformer-based models in capturing long-range dependencies, Transformers have recently demonstrated excellent performance in COD tasks. For example, FSPNet [29] employs ViT [30] as the encoder to capture global contextual information. FPNet [31] and FSNet [32] utilize PVT [33] and Swin Transformer [34], respectively, to extract globally representative features from input images for camouflaged object detection. Despite their strength in capturing global dependencies, Transformer-based architectures exhibit inherent limitations in modeling local patterns and preserving fine-grained details. The reliance on long-range interactions often comes at the expense of spatial precision, which is particularly detrimental for the accurate localization of camouflaged objects. To address these challenges, this study introduces a hybrid framework that integrates CNN and Transformer encoders, coupled with a novel multi-scale feature fusion mechanism. This design aims to alleviate the semantic discrepancy between heterogeneous features, enhance the quality of initial representations, and leverage the complementary advantages of both architectures to achieve more robust camouflaged object detection.

### 2.2. Complementary Information Strategy

Due to the inherent scale and information diversity of features extracted by encoders, as well as the inevitable encoding of noise and blurred boundaries, the practical performance of COD is often suboptimal. To enhance feature representation, various methods have attempted to integrate complementary low- and high-level features using structures [35] such as skip connections [36], short-range links [37], and neighboring pathways [21]. Despite their effectiveness, these techniques are often biased toward transferring information from lower-resolution stages to higher ones, which may restrict semantic expressiveness and neglect the dependencies across features. To address this, Song et al. [32] proposed a Cross-layer Connection Decoder (CCD), which selectively integrates intermediate-level features via element-wise multiplication and cross-layer sampling to enable computationally efficient multi-scale interaction, generating an initial prediction map focused on key regions. Similarly, Sun et al. [16] introduced a Cascade Correction Decoder (CCD), which performs iterative multi-scale feature optimization through bottom-up aggregation and top-down correction via dual-path collaborative learning. Such complementary fusion of multi-level features effectively enhances model robustness and discrimination capability in COD. In addition, several works have demonstrated that emphasizing the disparity between foreground and background representations contributes to more precise target localization. For example, Zhang et al. [38] introduced the Bilateral Attention Module (BAM), which utilizes a dual-branch attention mechanism to improve the representation of discriminative features in boundary regions. The foreground branch suppresses background interference and reinforces the target object, while the background branch mitigates foreground artifacts, significantly improving the integrity of fine details. Mei et al. [39] introduced PFNet (Positioning and Focus Network), where a higher-level prediction map and its inverse are multiplied with current-layer features to generate foreground- and background-aware features, which are then fed into a context exploration block to detect heterogeneous interference in either region. However, these approaches primarily aim at reducing interference from the foreground or background independently, lacking an explicit mechanism to decouple and regulate the interaction between the two.

## 3. Method

### 3.1. Overall Architecture

**Figure 2:**
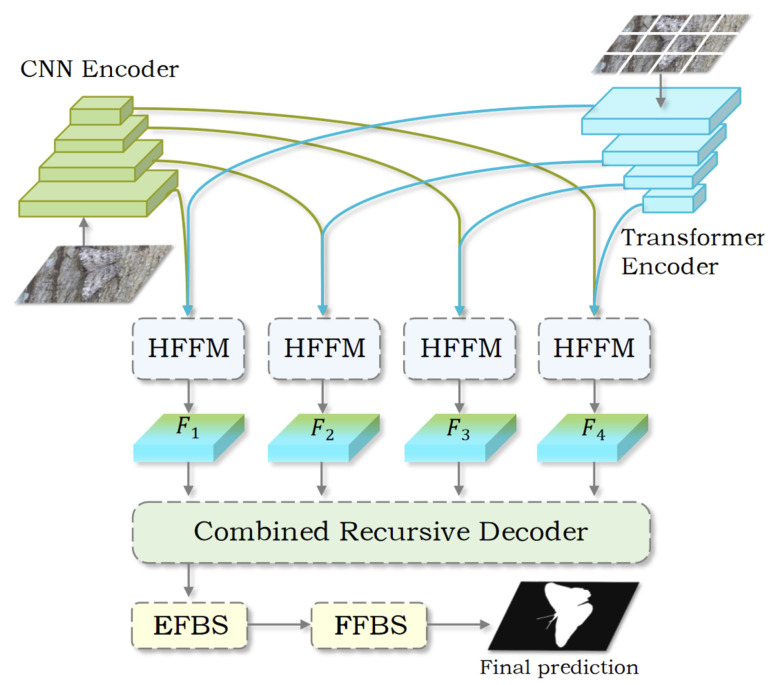
An overview of the proposed HRRNet: The input image is initially passed through a CNN-Transformer encoder to extract feature representations. These features are then fused using HFFM and passed through the CRD for progressive decoding. Finally, the boundaries are refined through the FBS module to achieve accurate prediction of camouflaged objects.

As shown in Figure 2, HRRNet consists of two stages. Stage 1 utilizes an encoder–decoder paradigm to extract meaningful representations and reconstruct camouflaged object structures, enhancing localization precision. Specifically, the RGB image $I\in R^{H\times W\times 3}$ is simultaneously processed through both CNN-based and Transformer-based encoders to extract multi-scale features, represented as $C_{i}$ and $T_{i}$, respectively, where i=1,2,…,4. To enhance global perception, an HFFM fuses the extracted CNN and Transformer features $C_{i}$ and $T_{i}$ through cross-level concatenation, producing the representations $F_{i}$ (i=1,2,…,4). The resulting features are subsequently passed to the CRD to integrate multi-scale information and output a coarse prediction. In stage 2, an FBS module is introduced. Guided by the coarse prediction, this module alternately focuses on foreground interiors/background boundary region to refine the object structure and boundary, enabling precise segmentation of complex camouflaged targets. To improve segmentation accuracy in complex backgrounds, the network jointly leverages fine-level texture cues and holistic semantic context, which facilitates precise boundary localization and effective object–background separation. We adopt a unified architecture and do not instantiate any background-specific branches or parameters.

### 3.2. Hybrid Feature Fusion Module

**Figure 3:**
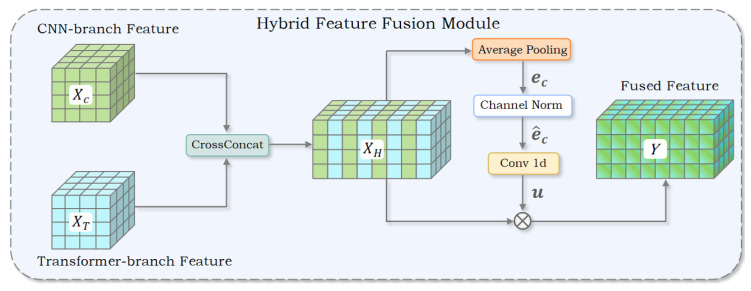
Schematic diagram of the HFFM. This module fuses CNN- and Transformer-encoded features through cross-stacking, channel normalization, and 1D convolution, enhancing semantic consistency and discriminative power.

In this study, features from CNN and Transformer streams are fused via a parallel integration scheme. Across the encoder’s four stages, feature maps undergo downsampling by factors of 4, 8, 16, and 32, with the number of channels set to 64, 128, 256, and 512, accordingly. The proposed HFFM comprises three key steps: (1) alternately stacking feature maps from the two branches to achieve cross-branch fusion, (2) applying L1-norm pooling to compute global features followed by channel-wise normalization, and (3) enhancing inter-channel dependencies through 1D convolution-based channel interaction. An overview of the internal design and components of the HFFM module is shown in Figure 3.

To facilitate the integration of channel-level cues from the CNN and Transformer branches, the module utilizes cross-connections to alternately arrange feature maps $X_{C}$ and $X_{T}$, thereby embedding pixel-wise semantics within the channel dimension. The feature map $X_{C}$ is placed at the even indices of $X_{T}$ with a stride of 2, while $X_{T}$ is inserted at the odd indices with the same stride. The specific formulation is as follows:

(1) $$\begin{array}{cc} X_{H}\left[0::2,\;:,\;:\right] & =X_{C}, \end{array}$$

(2) $$\begin{array}{cc} X_{H}\left[1::2,\;:,\;:\right] & =X_{T}, \end{array}$$

where $X_{C}$ and $X_{T}$ represent the feature maps from the CNN and Transformer branches, respectively, and $X_{H}$ is the fused feature map. Here, $X_{C},X_{T}\in R^{C\times H\times W}$, $X_{H}\in R^{2C\times H\times W}$. Subsequently, $L_{1}$ norm average pooling is applied to extract global contextual information from the fused feature map. Specifically, this pooling operation averages each channel across all spatial locations, yielding a fixed-size global feature vector $e\in R^{2C}$:

(3) $$e_{c}=\frac{1}{H\cdot W}\sum_{i=1}^{H}\sum_{j=1}^{W}X_{H}\left[c,i,j\right],$$

Based on this, motivated by ref. [40], we use channel-wise normalization to ensure numerical stability. The normalized vector $\hat{e}$ is computed as

(4) $${\hat{e}}_{c}=\frac{\sqrt{2C}\cdot e_{c}}{{∥e∥}_{2}}=\frac{\sqrt{2C}\cdot e_{c}}{{\left(\sum_{c=1}^{2C}e_{c}^{2}+\varepsilon \right)}^{1/2}}$$

Here, $e_{c}$ denotes the global descriptor for the *c*-th channel, while $X_{H}\left[c,i,j\right]$ represents the activation at spatial location (i,j) within the *c*-th channel of the fused feature map $X_{H}\in R^{2C\times H\times W}$. *H* and *W* correspond to the spatial dimensions, and *C* is the number of channels for each individual branch, either $X_{C}$ (from the CNN) or $X_{T}$ (from the Transformer). The normalized global context vector $\hat{e}$ lies in $R^{2C\times 1\times 1}$, where ${\left|e\right|}_{2}$ denotes the $L_{2}$-norm of the vector *e*, and ε is a small constant introduced to ensure numerical stability and prevent division by zero.

Finally, a 1D convolution is performed on the normalized context vector $\hat{e}$ to model local interactions across channels, generating a new channel-wise attention vector *u*:

(5) $$u=\mathrm{Conv}1D(\hat{e})$$

This attention vector *u* is used to recalibrate the original fused feature map $X_{H}$ through element-wise multiplication, resulting in the final output feature map *y*:

(6) $$y=X_{H}\cdot u$$

Here, $u\in R^{2C\times 1\times 1}$ denotes the learned channel-wise attention vector, while $y\in R^{2C\times H\times W}$ corresponds to the output feature map obtained after applying channel-specific modulation. $X_{H}$ is the intermediate feature representation obtained through cross-connection fusion of the CNN and Transformer branches.

### 3.3. Combined Recursive Decoder

**Figure 4:**
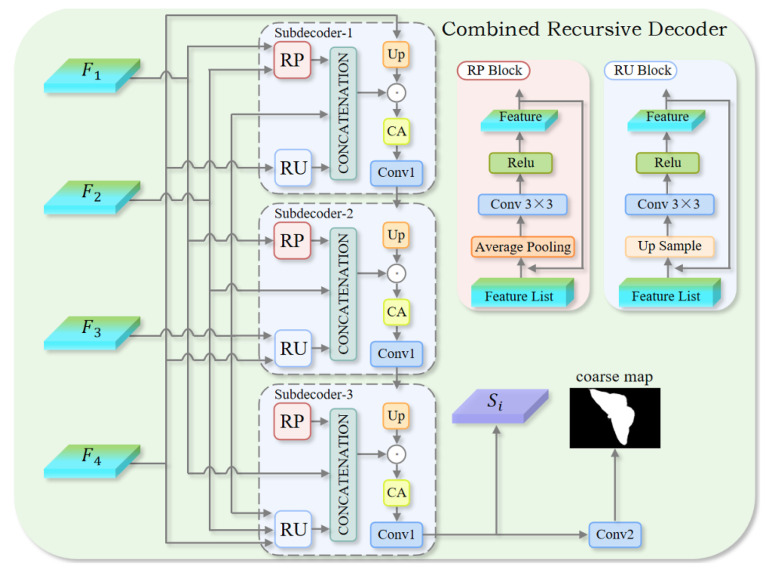
Schematic diagram of the CRD.

In previous studies, cascaded sub-decoders in decoders typically adopted the same strategy to sequentially fuse features from different levels. This repetitive approach overlooks the inherent differences among multi-scale features. To improve decoding performance, this paper proposes a sub-decoder that accounts for these discrepancies. By adopting a recursive method, it dynamically adjusts the feature fusion strategy, effectively addressing the variations between features at different levels and enhancing detection accuracy. The CRD module includes three recursive-based sub-decoders, as shown in Figure 4, to achieve the complete fusion of features $F_{i}$ (i=1,2,…,4).

The low-level features $F_{1}$ and $F_{2}$ are characterized by rich spatial resolution, which facilitates the extraction of detailed object boundaries. Conversely, the high-level features $F_{3}$ and $F_{4}$ encode stronger semantics, enabling effective discrimination between foreground and background. The CRD sub-decoders are designed to tailor their fusion mechanisms to the input feature scale, promoting stage-wise integration of hierarchical representations. Through the comprehensive multi-scale fusion of complementary low-level and high-level information, excellent camouflaged object detection results can be obtained. Specifically, each sub-decoder in the CRD module is tasked with feature fusion at a given scale. During feature fusion, the sub-decoder normalizes all scale information to ensure that the resulting feature maps remain reliable throughout the decoding process. Additionally, to ensure better detection results, the output from the preceding sub-decoder is passed to the subsequent sub-decoder for feature refinement. The entire decoding process is defined by the following formula:

(7) $$\begin{array}{c} mask_{m}={\mathrm{Des}}_{m}\left(F_{1},F_{2},F_{3},F_{4},mask_{m-1}\right), \end{array}$$

where ${mask}_{m}$ corresponds to the output produced by the *m*-th sub-decoder, and ${\mathrm{Des}}_{m}\left(\cdot \right)$ represents the operation performed by the *m*-th sub-decoder, with m=1,2,3. Within each sub-decoder, the Recursive Pooling (RP) module pools the input features, and then convolution and activation functions are applied to the pooled features. This process helps capture more spatial detail information at lower layers. The Recursive Upsampling (RU) module increases the feature map size through upsampling to recover detail information, followed by convolution and activation functions to further optimize these features. Each sub-decoder is fed with pooled features, spatially upsampled maps, weighted contextual references, and benchmark cues to guide feature alignment and refinement.

The pooled features and upsampled features are sequentially normalized using RP and RU, resulting in features $F_{3-m}^{\mathrm{mid}}$ and $F_{5-m}^{\mathrm{mid}}$, which match the shape of the benchmark features $F_{4-m}$. To construct the concatenated feature $F_{m}^{\mathrm{nor}}$, the benchmark features $F_{4-m}$ are combined with the normalized features $F_{3-m}^{\mathrm{mid}}$ and $F_{5-m}^{\mathrm{mid}}$ along the channel axis. Subsequently, the upsampled weighted reference features produced by the Upsampling module are employed to refine the concatenated representation. The refined features are then sequentially processed by the Channel Attention (CA) module and the Conv1 module to produce the final output of the *m*-th sub-decoder, denoted as ${\mathrm{mask}}_{m}$. At every stage, CRD forwards the upsampled prediction from the previous stage $U({\mathrm{mask}}_{m-1})$ as a mask guidance signal, which localizes object boundaries early and propagates that cue across scales. The entire process is defined as

(8) $$F_{k+1}^{\mathrm{mid}}=\mathrm{Cat}\left(D\left(F_{k}^{\mathrm{mid}}\right),F_{k+1}\right),k\in \left[1,2-m\right]$$

(9) $$F_{j}^{\mathrm{mid}}=\mathrm{Cat}\left(U\left(F_{j+1}^{\mathrm{mid}}\right),F_{j}\right),\;j\in \left[5-m,3\right]$$

(10) $$F_{m}^{\mathrm{nor}}=\mathrm{Cat}\left(D\left(F_{3-m}^{\mathrm{mid}}\right),F_{4-m},U\left(F_{5-m}^{\mathrm{mid}}\right)\right),$$

(11) $${\mathrm{mask}}_{m}={\mathrm{CC}}_{m}\left(F_{m}^{\mathrm{nor}}⊙U\left({\mathrm{mask}}_{m-1}\right)\right),$$

(12) $${\mathrm{coarse}}_{\mathrm{map}}=\mathrm{Conv}\left({\mathrm{mask}}_{3}\right),$$

where D(·) and U(·) represent downsampling and upsampling operations, ⊙ denotes element-wise multiplication, and Cat(·) represents channel-wise concatenation. The convolution operation ${\mathrm{CC}}_{m}\left(\cdot \right)$ consists of CA and Conv1 operations, performed by the *m*-th sub-decoder. Conv2 denotes the convolutional layer applied in the final stage to generate the coarse prediction map, which serves as the ultimate output of the CRD module. For the initialization, $F_{1}^{\mathrm{mid}}$ is set to 0, and for each sub-decoder, if 1>2−m, then *k* is an empty set. Similarly, if 5−m<3, *j* is similarly empty.

This mask-guided recursion detects and preserves boundary details of complex objects before producing the coarse map, and provides clean contours for subsequent FBS refinement.

### 3.4. Foreground–Background Selection Module

Camouflaged objects often reside in complex, texture-similar backgrounds. To mitigate the performance drop caused by subtle differences between the object and its surroundings, we introduce the FBS module. Rather than performing global background removal, FBS operates only in the ambiguous neighborhood of the object–background boundary, where confusion most often occurs. It alternates its focus between foreground (FFBS) interiors and the background boundary (EFBS) region, progressively refining the segmentation. By selectively refining informative areas, FBS enhances the model’s ability to recover the complete contour of camouflaged objects.

**Figure 5:**
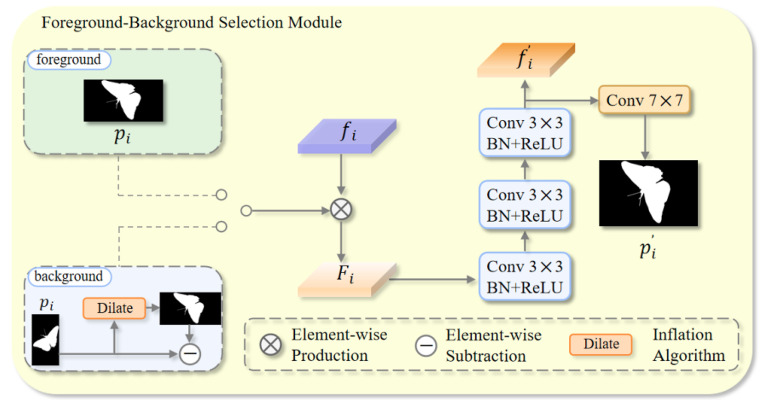
Schematic diagram of the FBS.

As shown in Figure 5, the FBS module takes two inputs: the current-level feature $f_{i}\in R^{C\times H\times W}$ and the coarse prediction map $p_{i}\in R^{1\times H\times W}$. It operates in two modes: FFBS emphasizes the foreground interiors by directly weighting the feature with $p_{i}$, so the network strengthens confident foreground regions and stabilizes the interior representation at the current level. EFBS emphasizes the background boundary belt around the object: we dilate the prediction and subtract the original prediction to highlight the narrow ring of pixels just outside the contour, and then use this ring to weight the feature. In effect, EFBS concentrates computation on the edge area, suppresses background leakage in texture-similar surroundings, and helps recover thin, high-curvature details (e.g., spikes, antennae, tapering tails). FFBS and EFBS are applied at different stages so that interiors are consolidated while boundaries are progressively refined. This process is summarized as

(13) $$M_{i}^{\prime }=D_{i}\left(p_{i}\right)-p_{i},$$

(14) $$F_{i}=f_{i}*\left\{\begin{array}{c} p_{i},\\ M_{i}^{\prime }, \end{array}\right.$$

where $D_{i}$(·) denotes the dilation operation (kernel size k = 7, iterations = 1), which is a dilation algorithm in computer vision used to emphasize regions in the background that are highly similar to the foreground boundary.

Next, a sequence of operations, including three 3×3 convolution layers, batch normalization, and ReLU activation functions, is applied to refine the fused features, producing the output $f_{i}^{\prime }\in R^{C\times H\times W}$. This refined output is then processed through a 7×7 convolution layer to generate $p_{i}\in R^{1\times H\times W}$. The entire process is expressed as

(15) $$\left\{\begin{array}{cc} f_{i}^{\prime } & =F_{3C_{3}BR}\left(F_{i}^{\prime }\right),\\ p_{i}^{\prime } & =F_{C_{7}}\left(f_{i}^{\prime }\right), \end{array}\right.$$

where $F_{3C_{3}BR}$ represents the sequence of three 3×3 convolution layers, batch normalization, and ReLU activations, and $F_{C_{7}}$ is a 7×7 convolution layer.

### 3.5. Loss Function

This paper utilizes binary cross-entropy (BCE) loss [41], $L_{\mathrm{BCE}}$, and intersection over union (IoU) loss [42], $L_{\mathrm{IoU}}$, to supervise the model’s predictions for camouflaged objects. This strategy differs from traditional loss formulations by assigning pixel-wise weights based on local contrast, which enhances the model’s focus on hard-to-segment regions. This mechanism is especially beneficial for COD tasks, where accurate boundary detection is essential. The formulas for these two losses are as follows:

(16) $$L_{\mathrm{BCE}}=-\frac{1}{N}\sum_{i=1}^{N}\left[y_{i}\log\left({\hat{y}}_{i}\right)+\left(1-y_{i}\right)\log\left(1-{\hat{y}}_{i}\right)\right],$$

(17) $$L_{\mathrm{IoU}}=1-\frac{\sum_{i=1}^{N}{\hat{y}}_{i}y_{i}}{\sum_{i=1}^{N}\left({\hat{y}}_{i}+y_{i}-{\hat{y}}_{i}y_{i}\right)},$$

where $y_{i}$ and ${\hat{y}}_{i}$ denote the ground truth label and predicted probability for the *i*-th pixel, respectively, while *N* represents the total number of pixels in the image. Therefore, the combined loss function, which incorporates the advantages of both, is defined as

(18) $$L_{\mathrm{total}}=\lambda_{1}L_{\mathrm{BCE}}+\lambda_{2}L_{\mathrm{IoU}},$$

where $\lambda_{1}$ and $\lambda_{2}$ are coefficients that control the relative weights of the two loss functions, and are typically set to 1 by default, based on empirical observations. Through this loss function, we ensure not only pixel-level classification accuracy but also improve the structural consistency and spatial alignment of the predicted masks, thus more effectively addressing the challenges in camouflage object detection tasks.

## 4. Experiments and Results

### 4.1. Experiment Settings

#### 4.1.1. Datasets

HRRNet is evaluated on four standard COD benchmarks: CHAMELEON [43], CAMO [23], COD10K [24], and NC4K [25]. CHAMELEON consists of 76 labeled images, all used for testing. CAMO contains 1250 samples, with a training/testing split of 1000/250. COD10K offers a broad range of camouflaged object types with 3040 training and 2026 testing images. NC4K contributes 4121 test images for further performance validation. In this study, the proposed HRRNet is trained on the 1000 images from the CAMO-Train subset and the 3040 images from the COD10K-Train subset.

The datasets used in this work include several representative object categories whose boundary geometries differ markedly, covering various geometric challenges. For example, insects (filamentary limbs/antennae; high aspect ratio and multi-branch structures), fishes (serrated/spiny contours with high-curvature segments), reptiles (elongated, tapering tails and long, narrow parts), mammals (smooth closed silhouettes under texture-similar backgrounds and touching boundaries), and seahorses (slender bodies with curled tails and multi-instance proximity).

#### 4.1.2. Implementation Details

The training of HRRNet is conducted using PyTorch 2.3.0 on an NVIDIA GTX 4090 GPU (24 GB). Four different backbones are employed: ResNet50, Res2Net50, Swin Transformer, and PVT. Optimization is performed with the Adam optimizer initialized at a learning rate of $5\times 10^{-5}$, which is gradually reduced using a polynomial decay schedule with an exponent of 0.9. Input images are uniformly resized to 384×384 prior to training. In accordance with previous works such as SINet [24] and FSPNet [29], data augmentation techniques, including horizontal flipping, random cropping, and color enhancement, are applied. The model is trained over 50 epochs with a batch size of 12.

#### 4.1.3. Evaluation Metrics

For performance evaluation, we use four standard metrics commonly employed in camouflaged object detection (COD) tasks: the structure measure ($S_{\alpha }$) [44], enhanced-alignment measure ($E_{\phi }$) [45], weighted F-measure ($F_{\beta }^{w}$) [46], and mean absolute error (MAE) [47]. $S_{\alpha }$ measures the structural similarity between the prediction and the ground truth by combining region-aware and object-aware components, thereby reflecting overall shape consistency and indirectly the integrity of object contours. $E_{\phi }$ (enhanced-alignment measure) evaluates the alignment between the prediction and ground truth by jointly considering local pixel agreement and global image-level statistics, capturing both global and local coherence. $F_{\beta }^{w}$ is a spatially weighted F-measure that re-weights false positives/negatives according to their distance to the ground-truth mask, placing larger penalties on confident errors far from the object while still accounting for boundary discrepancies; this yields a balanced assessment of interiors and boundary regions. MAE is the mean absolute error between continuous maps, penalizing deviations across the entire image. In general, higher $S_{\alpha }$, $E_{\phi }$, $F_{\beta }^{w}$, and lower MAE indicate superior COD performance.

### 4.2. Comparison with State of the Art

We evaluate the proposed HRRNet by benchmarking it against recent state-of-the-art approaches on four representative datasets to demonstrate its effectiveness. These methods include MGL [47], C2FNet [48], SINet-v2 [21], ZoomNet [26], FEDER [8], DGNet [18], FSPNet [29], Camouflageator [9], VSSNet [11], VSCode [12], FocusDiffuser [13], CamoFormer [22], EPFDNet [10], SegMar [49], LSR [25], FAPNet [50], SENet [51], and MCRNet [52]. To ensure consistency in evaluation, we distinguish between CNN- and Transformer-based backbones, and utilize prediction maps released by the original authors or obtained from official open-source implementations.

#### 4.2.1. Quantitative Evaluation

**Figure 6:**
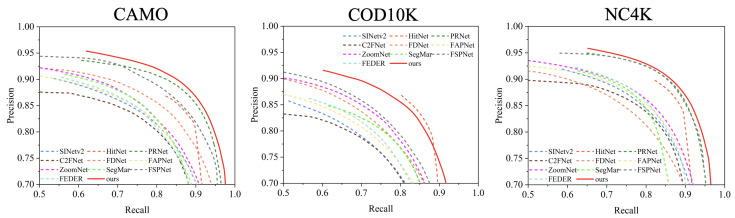
Precision–recall curves on three common COD datasets. Best viewed by zooming in for details.

**Figure 7:**
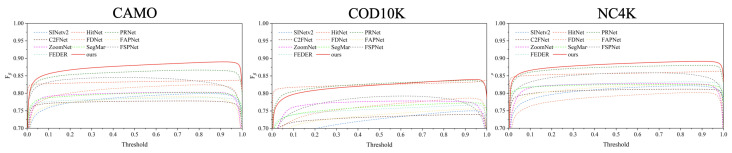
$F_{\beta }$ curves on three common COD datasets. Best viewed by zooming in for details.

As summarized in Table 1, the proposed HRRNet achieves consistent improvements over all the compared methods across four standard metrics. To thoroughly assess its effectiveness, we experiment with different configurations involving four widely adopted backbone encoders. Particularly on the CHAMELEON dataset, HRRNet outperforms all the baselines on every metric, with its $S_{\alpha }$ surpassing the runner-up by more than 1.0%. On the CAMO dataset, HRRNet attains an $S_{\alpha }$ score of 0.883, which is 1.0% higher than VSSNet (based on Swin Transformer) and 5.4% higher than Camouflageator (based on ResNet backbone). Despite the inherent challenges posed by the COD10K and NC4K datasets—known for their complex scenes and difficult object boundaries—HRRNet exhibits strong performance, validating the proposed complementary fusion and refinement designs. Furthermore, the PR and F-measure curves in Figure 6 and Figure 7 demonstrate that our method (red ‘Ours’ curves) consistently outperforms the competing approaches across all confidence thresholds.

Beyond the four standard COD metrics ($S_{\alpha }$, $E_{\phi }$, $F_{\beta }^{w}$, MAE), we also compare the number of parameters (Params, M), computational complexity (FLOPs, G), and runtime throughput (FPS, images per second) to provide a more comprehensive assessment. Params reflects the model size/capacity and memory footprint, FLOPs quantifies the hardware-agnostic computation per image (lower is more efficient), and FPS captures the end-to-end inference speed. As shown in Table 2, our HRRNet variants maintain moderate Params and FLOPs while achieving real-time FPS, yielding a favorable accuracy–efficiency trade-off compared with recent CNN/Transformer baselines.

#### 4.2.2. Qualitative Evaluation

**Figure 8:**
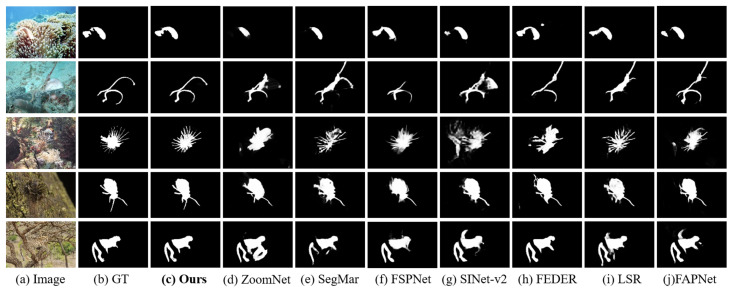
Visual comparisons of the proposed HRRNet and other seven state-of-the-art methods.

A visual comparison is illustrated in Figure 8, showcasing the performance of HRRNet against seven leading SOTA methods. The examples, selected from all four test datasets, represent a variety of complex environments such as underwater, land-based, and forested scenes. As observed from the prediction results, our method consistently yields more precise object boundaries and more complete object structures across various scenes. Specifically, for objects with fine-grained details and complex contours (e.g., the scorpionfish in the third row and the beetle in the fourth row of Figure 8), HRRNet is capable of delineating the object edges more accurately, preserving intricate structures such as antennae and spines. In contrast, competing methods such as ZoomNet and SINet-v2 often fail to capture these subtle details, exhibiting issues like missing parts and false predictions. In cases where the objects share high color and texture similarity with the background (e.g., the marine fish in the first row and the cheetah in the fifth row), HRRNet demonstrates superior discrimination, successfully segmenting the object with more complete contours while effectively filtering out occluding background elements. Methods solely based on Transformer or CNN backbones, such as FSPNet and LSR, tend to produce blurred segmentation results and suffer from background–object confusion. Moreover, in complex scenes containing multiple closely packed objects (e.g., the seahorses in the second row), our method is able to accurately reveal the spatial relationships and biological shapes of individual instances, outperforming other models in terms of structural integrity and object separation.

#### 4.2.3. Effectiveness Comparison of the HFFM and Dual-Encoder Strategy

To verify the impact of both the HFFM and the dual-branch encoder architecture on COD performance, a set of controlled ablation studies was conducted, with the detailed results shown in Table 3. Specifically, we compare three different model configurations: (1) a single-perception model using dual CNN encoders, (2) a global modeling model using dual Transformer encoders, and (3) a hybrid encoder model retaining both the CNN and Transformer encoders but with the HFFM module removed. These configurations are intended to assess the design rationale and performance contributions of our proposed method, focusing on network modeling capabilities, feature fusion strategies, and overall synergy. The experimental results reveal that both the dual-CNN and dual-Transformer encoder models exhibit performance limitations in handling camouflaged objects. The former lacks sufficient global perception capability, making it difficult to capture semantic consistency across large-scale camouflaged regions. In contrast, the latter, while capable of modeling long-range dependencies, falls short in representing local texture details, resulting in blurred boundaries and loss of object details. Furthermore, when the HFFM module is replaced by simple convolutional layers or naive feature concatenation, the model performance degrades significantly. This indicates that merely combining features from different encoders is insufficient for effective complementary learning. The HFFM module mitigates semantic discrepancies through channel alignment, normalization, and a strong interaction mechanism, thereby enhancing the consistency and discriminability of the fused features and ensuring the full exploitation of multi-scale and structural information. Therefore, both the dual-encoder design and the HFFM module are indispensable to the overall performance improvements of the model. Their synergy forms a solid foundation for subsequent multi-scale decoding and boundary refinement stages.

#### 4.2.4. Effectiveness Comparison of Combined Recursive Decoder

To comprehensively assess the contribution of the CRD, we carried out ablation comparisons, the results of which are detailed in Table 4. In this experiment, the CRD module was removed and replaced with a conventional decoder that does not employ recursive pooling, recursive upsampling, or recursive aggregation. We then conducted a thorough comparison with the complete model. It is evident from the results that excluding the CRD module significantly compromises segmentation performance, largely because standard decoders lack the capacity to effectively integrate multi-scale features with varying semantic and spatial characteristics. As a result, the fusion process fails to effectively integrate spatial details with semantic information. In contrast, the CRD module incorporates a recursive feature refinement strategy that effectively captures and enhances the spatial structure of target regions. The recursive design of the CRD module enables dynamic adjustment of multi-scale feature weights, leading to improved structural coherence and more precise boundary extraction. This makes the CRD an essential component for enhancing the overall reliability and detection accuracy of the proposed model. Its contribution to multi-scale integration and the preservation of structural details is essential for achieving high-quality segmentation, especially in challenging scenarios.

#### 4.2.5. Effectiveness Comparison of Foreground–Background Selection Module

To further investigate the contributions of FFBS and EFBS within the proposed FBS module, we conducted additional ablation experiments, as summarized in Table 5. To assess the individual contribution of each submodule, we conducted ablation experiments by independently removing the FFBS and EFBS components and analyzing the resulting performance changes. The results reveal that excluding the FFBS branch substantially impairs the model’s capacity to capture fine-grained details of the camouflaged object’s core structure. This underscores the effectiveness of the foreground-oriented strategy in enhancing salient object representation while suppressing background noise. Similarly, excluding the EFBS branch leads to a noticeable drop in boundary accuracy and detail preservation, indicating that attention to background edge regions is equally essential for mitigating foreground–background confusion and improving contour precision. Therefore, FFBS and EFBS together constitute a complete and complementary feature optimization framework. Accurate delineation of subtle structures is achieved through the dual-selection mechanism, which markedly enhances the model’s resilience and generalization performance in complex camouflaged environments.

#### 4.2.6. Qualitative Ablation Comparison

**Figure 9:**
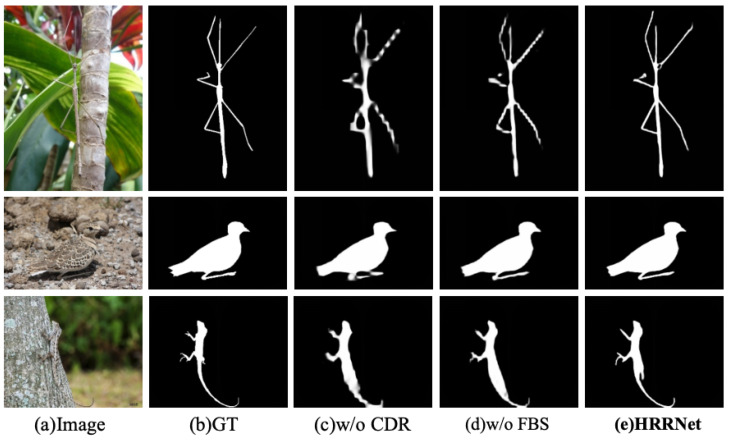
Qualitative ablation comparison. Columns (**left**→**right**): (**a**) Image, (**b**) GT, (**c**) w/o CRD, (**d**) w/o FBS, (**e**) HRRNet.

Beyond the quantitative gains reported in Table 4 and Table 5, Figure 9 provides side-by-side visual comparisons under two ablation settings. Removing the CRD leads to broken or over-smoothed shapes and missing thin parts (top: stick insect legs; bottom: lizard tail), indicating that recursive pooling/upsampling and aggregation are crucial to reconcile multi-scale semantics with spatial details. In contrast, removing the FBS degrades boundary localization and increases foreground–background confusion (e.g., edge fraying around the insect skeleton and leakage along the lizard silhouette), confirming the benefit of alternating foreground/background selection. The complete HRRNet preserves slender structures and sharp, clean contours across diverse scenes, aligning with the trends in $S_{\alpha }$/$E_{\phi }$/$F_{\beta }^{w}$/MAE.

## 5. Conclusions and Future Work

This work addresses a central challenge in camouflaged object detection (COD): integrating global semantics while preserving fine local structure. We propose HRRNet, a hybrid architecture that combines CNNs and Transformers. HRRNet is organized around three modules. The HFFM aligns and fuses heterogeneous CNN/Transformer features via cross-stacked fusion and channel-wise interaction, strengthening the joint representation. The CRD reconciles multi-scale features and reinforces object shape integrity during decoding. The FBS alternates attention between foreground structures and background edges, sharpening boundaries and reducing uncertainty in fine-grained predictions. Extensive experiments on CHAMELEON, CAMO, COD10K, and NC4K show state-of-the-art performance. On COD10K, HRRNet achieves an S-measure of 0.869 and MAE of 0.021, improving upon the Transformer baseline CamoFormer by 0.7% and the CNN baseline SINet-v2 by up to 1.5%, while reducing the error by 0.006. Ablation studies corroborate the effectiveness of each module and their complementary benefits when combined. This study has limitations. (i) Training and evaluation focus on static images; video and real-time COD remain to be validated. (ii) The recursive decoding and multi-level fusion that aid detail recovery introduce additional computation and latency compared with the lightest models, which may constrain edge deployment. (iii) Performance can degrade under extreme conditions (very low illumination, heavy occlusion, highly cluttered backgrounds). (iv) Reliance on pixel-level ground truth may limit generalization in domains with scarce or noisy annotations. Future work will explore lightweight module designs and real-time variants, robustness under extreme conditions, and self/weakly supervised learning. We also plan to investigate generalization to few-shot settings and video-based camouflage detection.

## Figures and Tables

**Table 1:** Comparisons of state-of-the-art methods on COD datasets is presented, with the top three results highlighted in red, green, and blue, respectively, where ↑ denotes that a higher score indicates better performance, and ↓ denotes that a lower score indicates better performance.

Method	Year	Backbone	CAMO-Test				COD10K-Test				NC4K				CHAMELEON			
			$S_{\alpha }↑$	$E_{\phi }↑$	$F_{\beta }^{\omega }↑$	M↓	$S_{\alpha }↑$	$E_{\phi }↑$	$F_{\beta }^{\omega }↑$	M↓	$S_{\alpha }↑$	$E_{\phi }↑$	$F_{\beta }^{\omega }↑$	M↓	$S_{\alpha }↑$	$E_{\phi }↑$	$F_{\beta }^{\omega }↑$	M↓
MGL [47]	2021-CVPR	ResNet50	0.775	0.812	0.736	0.088	0.814	0.852	0.711	0.035	0.833	0.867	0.782	0.052	0.893	0.917	0.833	0.031
$C^{2}$FNet [48]	2021-IJCAI	Res2Net50	0.796	0.854	0.762	0.080	0.813	0.890	0.723	0.036	0.838	0.897	0.765	0.049	0.888	0.935	0.828	0.032
SINet-v2 [21]	2022-TPAMI	Res2Net50	0.820	0.882	0.782	0.070	0.815	0.887	0.718	0.037	0.847	0.903	0.805	0.048	0.888	0.942	0.835	0.030
ZoomNet [26]	2022-CVPR	ResNet50	0.820	0.877	0.794	0.066	0.838	0.888	0.766	0.029	0.853	0.896	0.818	0.043	0.902	0.943	0.864	0.023
FEDER [8]	2023-CVPR	ResNet50	0.807	0.873	0.785	0.069	0.823	0.900	0.740	0.032	0.846	0.905	0.817	0.045	0.894	0.947	0.855	0.028
DGNet [18]	2023-MIR	EfficientNet	0.839	0.901	0.806	0.057	0.822	0.896	0.728	0.033	0.857	0.911	0.814	0.042	0.890	0.938	0.834	0.029
FSPNet [29]	2023-CVPR	ViT	0.857	0.899	0.830	0.050	0.851	0.895	0.769	0.026	0.879	0.915	0.843	0.035	0.908	0.943	0.867	0.023
Camouflageator [9]	2024-ICLR	ResNet50	0.829	0.891	0.805	0.066	0.843	0.920	0.763	0.028	0.869	0.922	0.835	0.041	0.903	0.952	0.863	0.026
VSSNet [11]	2024-TII	PVTv2	0.873	0.939	0.844	0.043	0.873	0.941	0.805	0.021	0.889	0.942	0.855	0.030	-	-	-	-
VSCode [12]	2024-CVPR	Swin-S	0.873	0.926	0.861	0.043	0.873	0.935	0.818	0.021	0.889	0.936	0.870	0.030	-	-	-	-
FocusDiffuser [13]	2024-ECCV	ViT	0.869	0.931	0.842	0.043	0.863	0.934	0.785	0.024	0.882	0.933	0.840	0.032	-	-	-	-
CamoFormer [22]	2024-TPAMI	Swin-B	0.876	0.935	0.832	0.043	0.862	0.932	0.772	0.024	0.888	0.941	0.840	0.031	0.891	0.953	0.829	0.026
EPFDNet [10]	2025-IVC	Res2Net50	0.817	0.886	0.757	0.068	0.815	0.894	0.700	0.033	0.844	0.906	0.780	0.044	-	-	-	-
SENet [51]	2025-TIP	ViT	-	-	-	-	0.865	0.925	0.780	0.024	0.889	0.933	0.843	0.032	-	-	-	-
MCRNet [52]	2025-IJCV	Swin	0.854	0.915	0.847	0.054	0.854	0.924	0.807	0.026	0.875	0.930	0.857	0.036	-	-	-	-
HRRNet	Ours	Swin-Res2Net	0.876	0.928	0.843	0.043	0.867	0.932	0.785	0.023	0.888	0.933	0.845	0.032	0.911	0.948	0.863	0.023
HRRNet	Ours	Swin-ResNet	0.874	0.926	0.842	0.045	0.866	0.931	0.783	0.023	0.886	0.931	0.847	0.032	0.910	0.948	0.862	0.024
HRRNet	Ours	PVT-Res2Net	0.883	0.935	0.853	0.041	0.869	0.941	0.797	0.021	0.891	0.942	0.855	0.030	0.918	0.959	0.869	0.021
HRRNet	Ours	PVT-ResNet	0.882	0.932	0.849	0.042	0.863	0.937	0.795	0.021	0.889	0.941	0.857	0.031	0.914	0.956	0.867	0.023

“-” denotes that the author does not provide corresponding results.

**Table 2:** Efficiency analysis of our HRRNet and multiple COD methods.

	MGL [47]	$C^{2}$FNet [48]	ZoomNet [26]	FEDER [8]	SegMar [29]	FSPNet [49]	HRRNet (Swin-R2N)	HRRNet (Swin-R50)	HRRNet (PVT-R2N)	HRRNet (PVT-R50)
Parameters	63.60	28.41	32.38	37.37	55.62	273.79	63.16	62.53	60.24	59.62
FLOPs (G)	553.94	26.17	203.50	23.98	33.65	288.31	36.67	35.80	35.06	34.28
FPS	5.18	109.67	14.10	119.68	85.29	10.13	78.42	80.17	82.15	83.92

**Table 3:** Ablation studies on the HFFM module and dual-encoder strategy, where ↑ denotes that a higher score indicates better performance, and ↓ denotes that a lower score indicates better performance.

Method	CAMO-Test				COD10K-Test				NC4K				CHAMELEON			
	$S_{\alpha }↑$	$E_{\phi }↑$	$F_{\beta }^{\omega }↑$	M↓	$S_{\alpha }↑$	$E_{\phi }↑$	$F_{\beta }^{\omega }↑$	M↓	$S_{\alpha }↑$	$E_{\phi }↑$	$F_{\beta }^{\omega }↑$	M↓	$S_{\alpha }↑$	$E_{\phi }↑$	$F_{\beta }^{\omega }↑$	M↓
CNN-CNN	0.825	0.897	0.798	0.056	0.823	0.907	0.760	0.030	0.861	0.913	0.825	0.041	0.901	0.938	0.855	0.028
Trans-Trans	0.859	0.918	0.836	0.043	0.844	0.923	0.774	0.026	0.879	0.924	0.836	0.036	0.911	0.946	0.859	0.027
w/o HFFM	0.876	0.928	0.841	0.046	0.861	0.927	0.788	0.025	0.883	0.930	0.841	0.033	0.912	0.952	0.860	0.024
HRRNet	0.883	0.935	0.853	0.041	0.869	0.941	0.797	0.021	0.891	0.942	0.855	0.030	0.918	0.959	0.869	0.021

**Table 4:** Ablation studies on Combined Recursive Decoder, where ↑ denotes that a higher score indicates better performance, and ↓ denotes that a lower score indicates better performance.

Method	CAMO-Test				COD10K-Test				NC4K				CHAMELEON			
	$S_{\alpha }↑$	$E_{\phi }↑$	$F_{\beta }^{\omega }↑$	M↓	$S_{\alpha }↑$	$E_{\phi }↑$	$F_{\beta }^{\omega }↑$	M↓	$S_{\alpha }↑$	$E_{\phi }↑$	$F_{\beta }^{\omega }↑$	M↓	$S_{\alpha }↑$	$E_{\phi }↑$	$F_{\beta }^{\omega }↑$	M↓
w/o CRD	0.823	0.894	0.785	0.060	0.815	0.903	0.742	0.033	0.855	0.916	0.790	0.043	0.886	0.922	0.813	0.034
HRRNet	0.883	0.935	0.853	0.041	0.869	0.941	0.797	0.021	0.891	0.942	0.855	0.030	0.918	0.959	0.869	0.021

**Table 5:** Ablation studies on Foreground–Background Selection module, where ↑ denotes that a higher score indicates better performance, and ↓ denotes that a lower score indicates better performance.

Method	CAMO-Test				COD10K-Test				NC4K				CHAMELEON			
	$S_{\alpha }↑$	$E_{\phi }↑$	$F_{\beta }^{\omega }↑$	M↓	$S_{\alpha }↑$	$E_{\phi }↑$	$F_{\beta }^{\omega }↑$	M↓	$S_{\alpha }↑$	$E_{\phi }↑$	$F_{\beta }^{\omega }↑$	M↓	$S_{\alpha }↑$	$E_{\phi }↑$	$F_{\beta }^{\omega }↑$	M↓
w/o FFBS	0.879	0.931	0.846	0.045	0.864	0.933	0.792	0.023	0.887	0.935	0.846	0.033	0.915	0.954	0.863	0.023
w/o EFBS	0.881	0.933	0.850	0.043	0.866	0.938	0.795	0.023	0.890	0.939	0.852	0.031	0.916	0.958	0.867	0.021
HRRNet	0.883	0.935	0.853	0.041	0.869	0.941	0.797	0.021	0.891	0.942	0.855	0.030	0.918	0.959	0.869	0.021

## Data Availability

The data presented in this study are openly available in https://github.com/DengPingFan/SINet/ (accessed on 25 July 2025), reference [24].
